# Prostaglandin D_2_ production in FM55 melanoma cells is regulated by α-melanocyte-stimulating hormone and is not related to melanin production

**DOI:** 10.1111/j.1600-0625.2010.01098.x

**Published:** 2010-08

**Authors:** Mojgan Masoodi, Anna Nicolaou, Karl Gledhill, Lesley E Rhodes, Desmond J Tobin, Anthony J Thody

**Affiliations:** 1 Bradford School of Pharmacy, School of Life Sciences, University of BradfordBradford, UK; 2 Centre for Skin Sciences, School of Life Sciences, University of BradfordBradford, UK; 3 Dermatological Sciences, School of Translational Medicine, University of Manchester and Salford Royal NHS Foundation Hospital, Manchester Academic Health Sciences CentreManchester, UK; 4 School of Clinical and Laboratory Sciences, University of Newcastle upon TyneNewcastle upon Tyne, UK

**Keywords:** α-melanocyte-stimulating hormone, liquid chromatography electrospray tandem mass spectrometry, melanogenesis, pigment cells, prostaglandin D_2_

## Abstract

This study shows that prostaglandins in human FM55 melanoma cells and epidermal melanocytes are produced by COX-1. Prostaglandin production in FM55 melanoma cells was unrelated to that of melanin suggesting that the two processes can occur independently. α-Melanocyte-stimulating hormone, which had no effect on melanin production in FM55 cells, stimulated PGD_2_ production in these cells without affecting PGE_2_. While cAMP pathways may be involved in regulating PGD_2_ production, our results suggest that α-MSH acts independently of cAMP, possibly by regulating the activity of lipocalin-type PGD synthase. This α-MSH-mediated effect may be associated with its role as an immune modulator.

## Background

Melanocytes produce melanin and have a role in skin pigmentation (1,2). Cutaneous prostaglandins, such as PGE_2_ and PGF_2α_, may act as mediators in this process because they increase melanocyte dendricity and melanogenesis (3,4). It has been suggested that these prostaglandins arise from keratinocytes (4) but it is possible that they are also produced by melanocytes acting as autocrine factors in the pigmentary response.

However, prostaglandins have a wide range of biological activities, and while some such as PGE_2_ act as pro-inflammatory mediators (5), others such as PGD_2_ down-regulate immune responses (6). PGD_2_ is formed by prostaglandin D synthase (EC 5.3.99.2) (PGDS), an isoform of which, lipocalin-PGDS (L-PGDS), is expressed in pigment cells (7). Expression of L-PGDS is dependent upon microphthalmia-associated transcription factor (MITF) (7) which is activated via cAMP and is involved in regulating melanogenesis (1,8,9). Thus, it is possible that PGD_2_ production is associated with melanogenesis, and the two processes have a common regulatory pathway.

α-Melanocyte-stimulating hormone (α-MSH) regulates melanogenesis via the cAMP-coupled melanocortin 1 receptor (MC1R) expressed on melanocytes (10–12). This peptide is also a potent immunomodulator through its effects on MC1R expressing immune cells such as monocytes and macrophages (13). Because melanocytes are immunocompetent (14–16), they might also mediate immunomodulatory actions of α-MSH. Their production of prostaglandins and, specifically, PGD_2_ could therefore be associated with this function.

## Questions addressed

Is the production of prostaglandins related to that of melanin in pigment-producing cells, and is it regulated by α-MSH?

## Experimental design

Prostaglandins were measured in melanin-producing FM55 human melanoma cells (17) and in human epidermal melanocytes. FM55 cells were used as a model system to examine the relationship between prostaglandin and melanin production and the effect of α-MSH. Because their MC1R does not couple to cAMP FM55 cells do not produce melanin in response to α-MSH (18). Their use therefore allowed the possibility of dissociating prostaglandin production from that of melanin.

The lightly pigmented FM55 cells were established from metastatic melanoma nodules (Dr AF Kirkin, Danish Cancer Society, Copenhagen, Denmark). Human epidermal melanocytes were isolated from skin samples obtained with local ethics committee approval and informed consent from donors undergoing elective plastic surgery. Cell culture (19,20), eicosanoid analysis (21), stimulation and measurement of melanin (20,22) and COX-1/-2 protein expression (5) were performed as published; L-PGDS was measured using an immunometric kit (Appendix S1).

## Results

**Figure 1:**
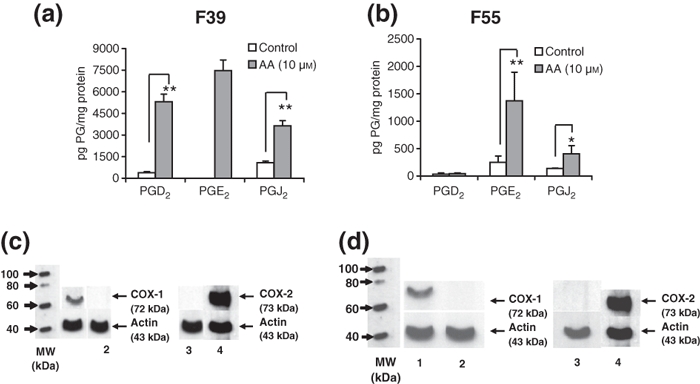
Sample profile of prostaglandins (PG) produced by human epidermal melanocytes F39 (a) and human melanoma FM55 (b) under resting conditions (Control) and following treatment with arachidonic acid (AA) (10 μm for 24 h). Expression of COX-1 and COX-2 proteins in human epidermal melanocytes F39 (c) and human melanoma FM55 cells (d) assessed by Western blotting analysis. MW: molecular weight markers; Lane 1: COX-1; Lane 2: COX-1+ COX-1 blocking peptide; Lane 3: COX-2; Lane 4: COX-2-positive control using FM3 hamster melanoma cells. Note: each antibody (i.e. COX-1 and COX-2) was independently carried on its own lane of the same gel. Data shown as mean ± SEM of *n* = 3 independent experiments. *P< 0.05 and **P < 0.005, comparing data to control.

PGD_2_ and PGE_2_ were the major prostaglandins identified in human epidermal melanocytes and FM55 melanoma cells (Fig. 1a, b). Lipidomic analysis did not confirm production of PGF_2α_ by FM55, as previously reported using a less specific radiometric approach (17). Western blotting analysis revealed that FM55 cells and melanocytes expressed the constitutive isoform of cyclooxygenase (COX-1) but not the inducible isoform COX-2 (Fig. 1c, d).

**Figure 2:**
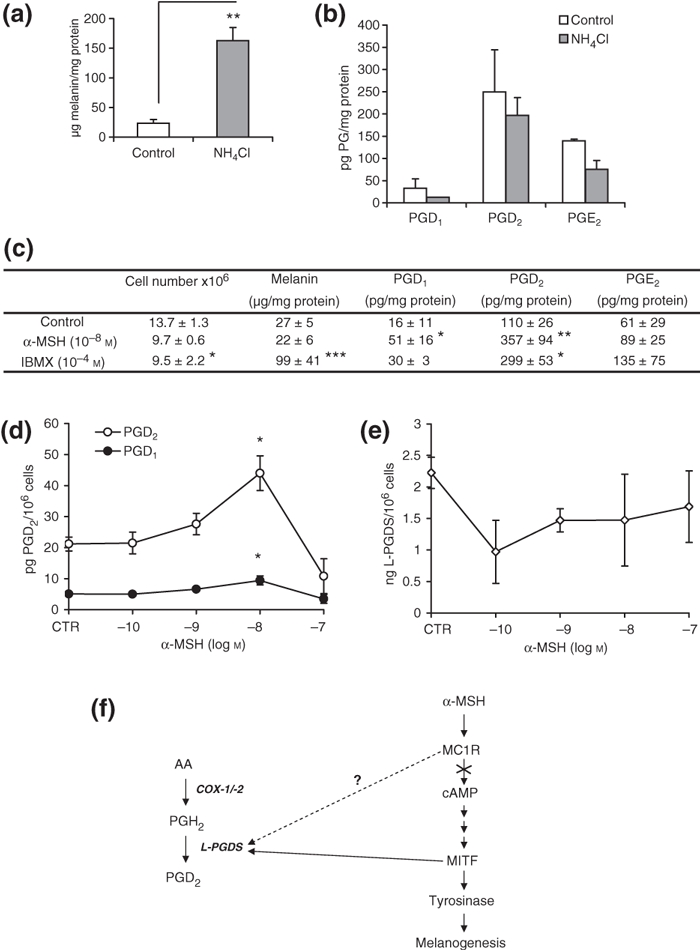
Prostaglandin (PG) and melanin production in FM55 human melanoma cells. The effect of NH_4_Cl (10 mm) and l-tyrosine (400 μm) on (a) melanogenesis and (b) prostaglandin production. (c) The effect of α-Melanocyte-stimulating hormone (α-MSH) (10^−8^ m) and IBMX (10^−4^ m) on cell number, levels of melanin, PGD_1_, PGD_2_ and PGE_2_, following 48-h treatment. Dose-dependent effect of α-MSH (10^−10^–10^−7^ m) on (d) PGD_1_ and PGD_2_ production and (e) lipocalin-prostaglandin D synthase expression. Data expressed as mean ± SEM of *n* = 3 independent experiments. *P < 0.05, **P < 0.01 and ***P < 0.001 comparing data to control (CTR). (f) Schematic outline of the major signalling pathways involved in melanin and PGD_2_ production. In FM55 cells, the MC1R does not couple to cAMP as indicated by the cross. As a consequence, α-MSH fails to stimulate melanin production, and the regulation of PGD_2_ production may be via a cAMP independent pathway as indicated by the dotted line. AA, arachidonic acid; PGH_2_, prostaglandin H_2_; MC1R, melanocortin 1 receptor; MITF, microphthalmia-associated transcription factor.

Increasing melanin production in FM55 had no effect on prostaglandin production (Fig. 2a, b); when prostaglandins were stimulated with arachidonic acid, melanin production was not affected (23 ± 6 and 24 ± 4 μg melanin/mg cell protein, before and after treatment, respectively).

α-MSH had no effect on PGE_2_ production in FM55 cells but increased PGD_2_ and PGD_1_ with no effect on melanin (Fig. 2c). 3-Isobutyl-1-methylxanthine (IBMX), which raises cAMP levels by inhibiting phosphodiesterase, increased the production of PGD_2_ and, in contrast to α-MSH, increased melanin production (Fig. 2c). As shown in Fig. 2d, the effect of α-MSH on PGD_2_ production was dose-related, the maximal increase occurring in response to 10^−8^mα-MSH, a dose within the physiological range of concentrations of α-MSH in the epidermis (2). At 10^−7^m, α-MSH reduced PGD_2_ to control levels. Similar changes were seen with PGD_1_. L-PGDS was present in FM55 melanoma cells, but protein levels were unaffected by α-MSH (Fig. 2e).

## Conclusion

It has been suggested that prostaglandins have a role in the pigmentary response (3,4). However, we found no such association between prostaglandin and melanin production in FM55 melanoma cells. This dissociation was further demonstrated in experiments with α-MSH. Although this peptide is melanogenic in human melanocytes via the MC1R (10,11), it fails to have this effect in FM55 cells (18), as confirmed here, yet it increased prostaglandin production. Thus, it would seem that in FM55 cells prostaglandins are produced as part of some non-pigmentary function.

PGD_2_ is a major prostaglandin in both epidermal melanocytes and FM55 cells. α-MSH modulated the production of PGD_2_ in a concentration-dependent manner in FM55, producing a bell-shaped dose response curve similar to that observed for melanin (10) and NO (15). As with many of its actions, it seems that α-MSH is a modulator rather than an outright stimulator.

α-MSH may act specifically to regulate PGD_2_ synthesis at the level of L-PGDS (Fig. 2f). This is supported by the concomitant stimulation on PGD_1_ but lack of effect on PGE_2_, indicating that α-MSH is not acting at the level of COX. Our findings indicate that α-MSH may affect the activity, but not expression of L-PGDS. Expression of L-PGDS is upregulated by MITF (7), which is under the control of the cAMP signalling pathway (23). This would explain the increase in PGD_2_ production observed in response to IBMX-dependent increased cAMP. It is unlikely that α-MSH acts in this way because the MC1R on FM55 cells does not couple to cAMP (18). We therefore propose that α-MSH acts independently of cAMP and activates L-PGDS rather than inducing its expression. Further studies using human epidermal melanocytes and melanoma cells with different degrees of pigmentation are needed to elucidate this effect of α-MSH and determine whether it is a common property of pigment-producing cells.

PGD_2_ can inhibit growth of human melanoma cells (24) and loss of L-PGDS expression may be important in allowing the tumor to avoid immune surveillance (25). The fact that PGD_2_ is a product of immune cells, such as Langerhans cells, mast cells and macrophages (26) emphasizes its importance as an immunomodulator. Melanocytes are another potential source of cutaneous PGD_2_; this together with the regulation of PGD_2_ production by α-MSH underlines their importance as mediators of immune responses in the skin.
