# Supplementary material for: Prostaglandin D2 production in FM55 melanoma cells is regulated by α-melanocyte-stimulating hormone and is not related to melanin production
Source: Exp Dermatol. 2010 Aug;19(8):751–3. doi: 10.1111/j.1600-0625.2010.01098.x (PMC3068286; doi:10.1111/j.1600-0625.2010.01098.x)
Supplement: Supplementary file 1 [file exd0019-0751-SD1.doc]

## Materials and Methods

**Materials**

RPMI 1640, Eagle’s minimal essential medium, L-glutamine, penicillin/streptomycin, trypsin/EDTA and phosphate buffered saline (PBS) were purchased from Gibco (Paisley, UK). Fetal calf serum (FCS) was obtained from Invitrogen (Paisley, UK). α-MSH was purchased from Bachem (St Helens, UK). Dimethyl sulphoxide (DMSO), sodium hydroxide (NaOH), citric acid, calcium chloride (CaCl_2_), ammonium chloride (NH_4_Cl), L-tyrosine, 3-isobutyl-1-methylxanthine (IBMX), synthetic melanin, HPLC grade glacial acetic acid and hydrochloric acid were purchased from Sigma (Poole, UK). All other HPLC-grade organic solvents were from Fisher Chemicals (Loughborough, UK). Prostaglandin standards were purchased from Cayman Chemicals (Ann Arbor, MI, USA). Solid phase extraction (SPE) cartridges (C18-E 500 mg, 6 ml) were purchased from Phenomenex (Macclesfield, UK).

**Cell Culture**

Human epidermal melanocytes (cells F39) were isolated from skin samples obtained with local ethics committee approval and informed consent from donors undergoing elective plastic surgery. The melanocytes were cultured in supplemented Eagle’s minimal essential medium as previously described [1]. FM55 human melanoma cells were maintained in RPMI 1640 containing 10% FCS and penicillin (50 IU per ml)/streptomycin (50μg per ml) as described previously [2]). All cultures were kept in a humidified atmosphere at 37^○^C, 5% CO_2_. FM3 hamster melanoma cells were also grown in RPM1 1640 (as described for FM55 and FM94 cells) and used as positive control for the expression of COX-2 protein.

**Prostaglandin production by melanocytes and melanoma cells**

**Prostaglandins at resting conditions**

Cells were seeded into T75 flasks and grown up to 80% confluence. The medium was removed, cells were washed in PBS to remove any remaining FCS-containing medium, and replaced with serum-free medium (10 ml). The cells were then incubated for a further 24 h. Incubation with serum-free media did not affect the ability of the cells to adhere to the cell culture flasks. At the end of this period the incubation medium was collected and stored in a polyethylene tube at -20°C awaiting prostaglandin analysis. The cells were collected and used for western analysis.

**Stimulation of prostaglandin production with arachidonic acid**

Cells (melanocytes and/or melanoma cells) were grown to 80% confluence and transferred to serum-free medium (10 ml) as described before (resting conditions). Prostaglandin production was stimulated with arachidonic acid (10 μΜ) (30 μl of a 1 μg/μl solution in ethanol in ethanol). Control cells (melanocytes and/or melanoma cells) were treated with the vehicle alone. The cells were then incubated at 37°C for 24 h. The medium was collected and stored at -20°C for prostaglandin analysis. The cells (control and treated) were collected and assessed for melanin production.

**Effect of melanogenesis on prostaglandin production**

FM55 melanoma cells were grown to 80% confluence and transferred to serum-free media as described before (resting conditions). Melanin production was increased by treating the cells with 10 mM NH_4_Cl and 400 μM L-tyrosine for 48 days as previously described [3]. At the end of the treatment period the medium was collected for prostaglandin analysis, and the cells were collected and used to assess melanin production.

**Effect of α-MSH and IBMX on prostaglandin production**

FM55 melanoma cells were grown to 80% confluence and transferred to serum free media as described before (resting conditions). The serum-free medium was supplemented with α-MSH (10^-8^ M) (stock solution prepared in PBS : glacial acetic acid, 50:50 v/v) or IBMX (10^-4^M) (stock solutions prepared in PBS) and the cells were incubated at 37°C for 48 h. Control experiments were run in parallel using the vehicle alone. The medium was collected and stored at -20^o^C for prostaglandin analysis whilst the cells were collected and used to assess melanin production. For the dose-dependent study, α-MSH was added to give a final concentration range of 10^-10^ -10^-7^M. At the end of the experiment the medium was used to estimate levels of prostaglandins and the cells were used to assess the expression of L-PGDS.

**Lipidomic analysis**

Lipidomic analysis of eicosanoids in the cell culture media was performed according to our published protocols [4, 5]. In brief: cell culture media from the different treatments (10 ml) were thawed and kept on ice. Methanol (1.77 ml) was then added to adjust the solution to 15% (v/v) methanol, followed by the addition of internal standard PGB_2_-*d*4 (40 μl of 1 ng/μl solution). The resulting solutions were then acidified with 0.1 M hydrochloric acid to pH 3.0 and immediately applied to pre-conditioned SPE cartridges. The cartridges were washed with 15% (v/v) methanol in water (20 ml) followed by 20 ml of water (20 ml) and hexane (10 ml); the lipid mediators were eluted in methyl formate (15 ml). The organic solvent was evaporated using a fine stream of nitrogen and the remaining residue was re-dissolved in ethanol (100 μl) stored at –20°C awaiting analysis.

Chromatographic analysis was performed on a C18 column (Luna 5 μ, Phenomenex (Macclesfield, UK) using a Waters Alliance 2695 HPLC pump coupled to an electrospray (ESI) triple quadrupole Quattro Ultima mass spectrometer (Waters, Elstree, Hertsfordshire, UK). Instrument control and data acquisition were performed using MassLynx^TM^ V4.0 software. The following multiple reaction monitoring (MRM) transitions were used for the assay of prostaglandins: PGD_1_ *m/z* 353>317; PGE_2_ *m/z* 351>271; PGD_2_ *m/z* 351>271; PGJ_2_ *m/z* 333>271. Results are expressed as pg prostaglandin / mg of protein based on calibration lines constructed with commercially available prostaglandin standards. Protein content was estimated using the BioRad protein assay kit with BSA as standard (BioRad, Hemel Hempstead, UK).

**Protein expression**

**Expression of COX-1 and COX-2**

The presence of COX-1 and COX-2 protein was assessed by immunoblotting in human epidermal melanocytes (passage 4), FM55 human melanoma cells and hamster melanoma cells FM3. Cells were then solubilized on ice using Laemli’s buffer in the presence of protease inhibitors (Sigma, Poole, UK) for 4 h. Protein concentration was measured using the modified Bradford assay (Bio-Rad, Hemel Hempstead, UK), and identical amounts of extracted protein samples (35 µg) were separated by sodium dodecyl sulfate-8% PAGE under reducing conditions. After electrophoretic separation, proteins were electroblotted onto polyvinylidene difluoride (PVDF) membranes (Immobilon, Millipore, Bedford, MA, USA), and blocked for 2 h at RT with 5% nonfat milk (Marvel Ltd., Merseyside, UK) in PBS.

The membranes were immunoprobed for 18 h at 4°C with antibodies against COX-1 (1:100) (goat polyclonal, Santa Cruz Biotechnology, CA, USA), COX-2 (1:75) (rabbit polyclonal; Cayman Chemicals, Ann Arbor, MI, USA) and actin (used as loading control) (1:250) (goat polyclonal, Santa Cruz Biotechnology, CA, USA). As a control for COX-1 the antibody was pre-absorbed with COX-1 blocking peptide (1:100) (Santa Cruz Biotechnology, CA, USA) for 1 h at room temperature before immunoprobing the membrane. The membranes were then incubated for 2 h at room temperature with a horseradish peroxidase-conjugated donkey anti-sheep/goat IgG Ab (1:600) (Serotec Ltd., Kidlington, Oxford, UK) or a horseradish peroxidase-conjugated donkey anti-rabbit IgG Ab (1:1000) (GE Healthcare, Chalfont St Giles, Buckinghamshire, UK) and developed by the Enhanced Chemiluminescence plus Western blot detection system kit, according to the manufacturer’s instructions (Amersham Biosciences Ltd., Little Chalfont, Buckinghamshire, UK). Results were visualized using photographic film (Kodak X-omat LS Film, Sigma, Dorset, UK).

**Expression of L-PGDS**

The effect of α-MSH on L-PGDS expression in FM55 cells was assessed using an immunometric EIA kit (Cayman, Ann Arbor, MI, USA) following the manufacturer’s instructions.

**Measurement of melanin production**

Melanin content in the cells was assessed by dissolving the cell pellets in 1M NaOH and measuring the absorbance of the resulting solution at 475 nm. Quantitation of melanin was based on calibration lines constructed with commercially available standard [3].

**Statistical analysis**

Data were analysed with SPSS 14.0 using one-way ANOVA applying Bonferroni’s correction for multiple comparisons. Unless otherwise stated, all results are compared to the corresponding non-treated controls. Statistical significance was accepted at the *p*<0.05 level.

**References**

1. Kauser S, Schallreuter KU, Thody AJ, Gummer C, Tobin DJ: Regulation of human epidermal melanocyte biology by beta-endorphin. J Invest Dermatol 2003;120(6):1073-80.

2. Ancans J, Tobin DJ, Hoogduijn MJ, Smit NP, Wakamatsu K, Thody AJ: Melanosomal pH controls rate of melanogenesis, eumelanin/phaeomelanin ratio and melanosome maturation in melanocytes and melanoma cells. Exp Cell Res 2001;268(1):26-35.

3. Hoogduijn MJ, Smit NP, van der Laarse A, van Nieuwpoort AF, Wood JM, Thody AJ: Melanin has a role in Ca2+ homeostasis in human melanocytes. Pigment Cell Res 2003;16(2):127-32.

4. Masoodi M, Mir AA, Petasis NA, Serhan CN, Nicolaou A: Lipidomic analysis of twenty-seven prostanoids and isoprostanes by liquid chromatography/electrospray tandem mass spectrometry. Rapid Commun Mass Spectrom 2008;22(2):75-83.

5. Masoodi M, Nicolaou A: Lipidomic analysis of twenty-seven prostanoids and isoprostanes by liquid chromatography/electrospray tandem mass spectrometry. Rapid Commun Mass Spectrom 2006;20(20):3023-9.
